# THE RELATIONSHIP BETWEEN THE CAREGIVING ENVIRONMENT, BURNOUT AND QUALITY OF LIFE AMONG HOME HEALTH AIDES

**DOI:** 10.1093/geroni/igad104.3657

**Published:** 2023-12-21

**Authors:** Sehyun Baek, Soobin Park, Oejin Shin, Sojung Park, Ahra Ko

**Affiliations:** Washington University in Saint Louis, St. Louis, Missouri, United States; Washington University in Saint Louis, St. Louis, Missouri, United States; Illinois State University, Normal, Illinois, United States; Washington University in Saint Louis, St. Louis, Missouri, United States; Yonsei University, Seoul, Republic of Korea

## Abstract

The well-being of home health aides (HHAs) is affected by the characteristics of their work environment. As South Korea’s population rapidly ages, there is an increasing demand for home aides. However, little is known about how the caregiving environment affects HHAs. Guided by the environment comfort model, we examined the association between care recipients’ home environment and HHA’s quality of life, focusing on how burnout mediates this relationship. Our data came from a national survey of home health aides in 2020 (N=981). We conducted an exploratory factor analysis to identify six factors related to the care environment in three dimensions: physical (1. space; 2. indoor/outdoor conditions), functional (3. home appliances; 4. heating/air conditioning), and psychological (5. satisfaction with the home environment; 6. relationships with care recipients and their families). We then used a path analysis to examine the relationship between these factors, burnout, and quality of life. Our findings show that safe indoor/outdoor conditions and positive relationships with care recipients and their families are associated with lower levels of burnout, leading to a higher quality of life (p< 0.05, respectively). This highlights the importance of considering both physical and psychological aspects of the caregiving environment to prevent burnout and improve the quality of life for HHAs, ultimately contributing to high-quality services for care recipients.

